# Mutualisms and Population Regulation: Mechanism Matters

**DOI:** 10.1371/journal.pone.0043510

**Published:** 2012-08-23

**Authors:** Shalene Jha, David Allen, Heidi Liere, Ivette Perfecto, John Vandermeer

**Affiliations:** 1 Department of Integrative Biology, University of Texas, Austin, Texas, United States of America; 2 Department of Biology, Middlebury College, Middlebury, Vermont, United States of America; 3 Department of Entomology, University of Wisconsin, Madison, Wisconsin, United States of America; 4 School of Natural Resources and Environment, University of Michigan, Ann Arbor, Michigan, United States of America; 5 Department of Ecology and Evolutionary Biology, University of Michigan, Ann Arbor, Michigan, United States of America; Centro de Investigación y de Estudios Avanzados, Mexico

## Abstract

For both applied and theoretical ecological science, the mutualism between ants and their hemipteran partners is iconic. In this well-studied interaction, ants are assumed to provide hemipterans protection from natural enemies in exchange for nutritive honeydew. Despite decades of research and the potential importance in pest control, the precise mechanism producing this mutualism remains contested. By analyzing maximum likelihood parameter estimates of a hemipteran population model, we show that the mechanism of the mutualism is direct, via improved hemipteran growth rates, as opposed to the frequently assumed indirect mechanism, via harassment of the specialist parasites and predators of the hemipterans. Broadly, this study demonstrates that the management of mutualism-based ecosystem services requires a mechanistic understanding of mutualistic interactions. A consequence of this finding is the counter intuitive demonstration that preserving ant participation in the ant-hemipteran mutualism may be the best way of insuring pest control.

## Introduction

Providing food for the world is fundamentally an ecological issue given that agricultural ecosystems follow the same ecological laws as all other ecosystems [1]. One ecological process that is frequently acknowledged as central to ecosystem organization is the formation of mutualisms. Mutualisms are ubiquitous in both natural and managed ecosystems and are especially integral to global agriculture, since crop species are often dependent on mutualistic interactions such as pollination [2], nitrogen fixation [3], and mycorrhizal associations [4]. A less advertised, but extremely important mutualism in agroecosystems is between ants and their hemipteran partners [5], [6], usually seen as detrimental to crops since hemipterans frequently reach pest status and threaten crop production [7], [8].

While hemipterans may appear to benefit from ant presence, the mechanism and degree of mutualistic interaction is widely contested [9]. In laboratory settings without predators, the direct benefit of ant presence has been demonstrated to be mediated by the removal of sooty mold which negatively affects hemipteran growth rates [10], [11]. In the presence of predators, however, it has long been argued that the indirect release from predation pressure is the primary driver of the ant-hemipteran mutualism [12], [13], [14], [15]. Assuming the latter, it is frequently presumed that hemipteran population control in agroecosystems necessitates elimination of ants.

**Figure 1:**
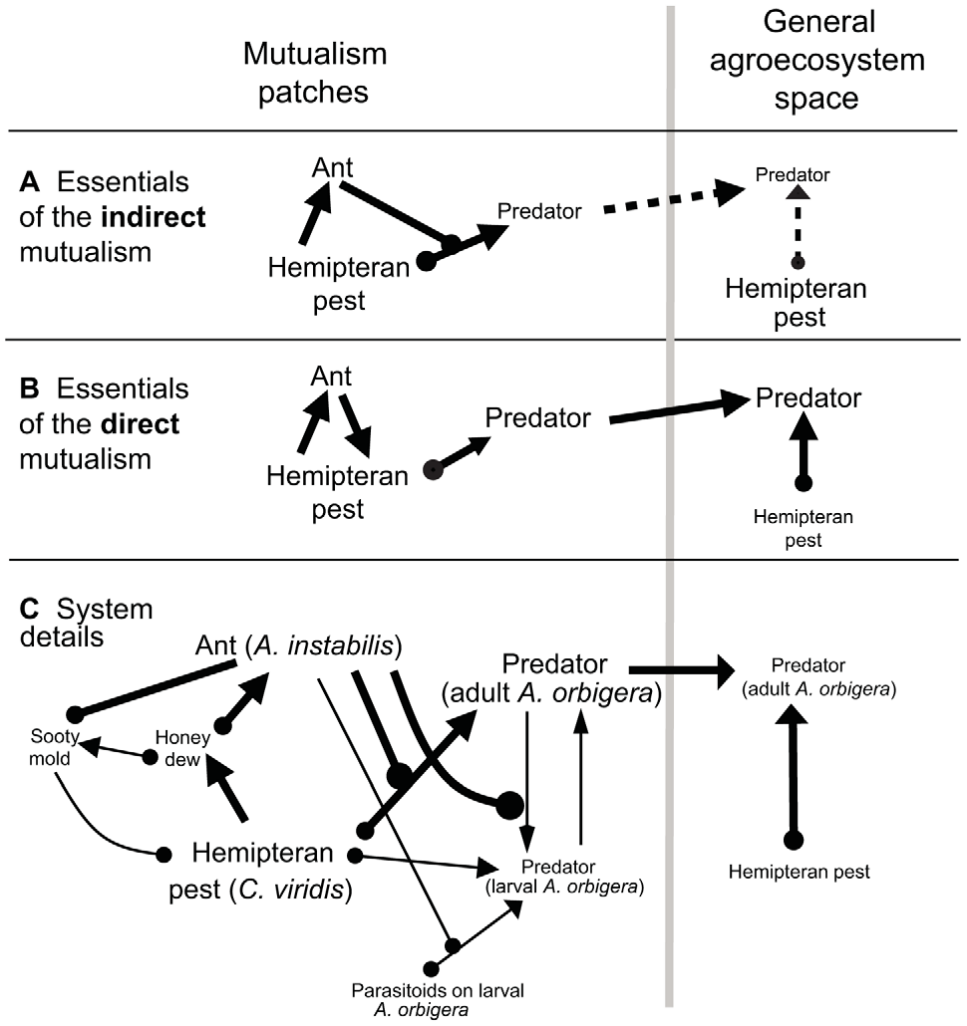
Diagrammatic representation of the two hypotheses describing the mutualism mechanism and details of the study system. (A) Indirect mutualism situation where the mutualism patches do not generate spatial reservoirs of the predator, and (B) direct mutualism situation, where the mutualism patches generate spatial reservoirs. Solid black lines indicate strong effects, and black dotted lines indicate weak effects. An arrow indicates a positive effect on the target organism, a circle indicates a negative effect on the target organism, and text size indicates relative population density. (C) The details of the study system, where bold lines refer to the generalized interactions and non-bold lines to the details of the particular study system. The direct effect (i.e., not trait-mediated) is that the ant either consumes sufficient honeydew to prevent sooty mold or removes the sooty mold directly. The indirect effect (i.e., trait-mediated) is the behavioral effect the ants have on the ability of the predator adults to lay eggs in the general area where the hemipteran pest is located and the ability of the predator adults to consume the hemipterans. The positive effect the ants have by protecting the larval predators against their parasitoids does not negate the negative effects the ant has on the adult predator through direct interference with its ability to eat and oviposit. The question as to whether the system is a direct mutualism (B) or an indirect mutualism (A) depends on the relative values of all the interactions in the system details (C).

If an alternate direct mechanism for the mutualism were operative, however, such a proposal could be counter-productive. Assume, as is usually the case, that ants and their mutualistic partners have an aggregated distribution and therefore are concentrated in patches within the agroecosystem space [16]. If, as is frequently assumed, ants provide the hemipterans protection from their specialist predators (i.e. indirect mutualism), then specialist predator populations will not be able to build up sufficiently large densities to operate as effective biological controls in the general agroecosystem space (Fig. 1A). However, if the mechanism of the mutualism is direct, that is, not through interference with predation but rather through improved hemipteran growth and reproduction, then the patches with the mutualism could generate larger densities of specialist predators, effectively becoming a spatial reservoir for the predator (Fig. 1B). In other words, an indirect mechanism of an ant-hemipteran mutualism would interfere with natural control of the pest (Fig. 1A) while a direct mechanism could, in some cases, enhance it (Fig. 1B). Thus, with regard to the ecosystem service of pest control, the mechanism of the mutualism matters.

Despite the ubiquity of ant-hemipteran mutualisms, the specific mechanism by which ant-tended patches support both specialist predator and hemipteran populations has rarely been investigated in the field. In this study, we examine the mechanism of mutualistic interaction between the green coffee scale, *Coccus viridis*, and the hemipteran-tending ant, *Azteca instabilis,* in a coffee growing region in Chiapas, Mexico. In this study system, we know that the main predator of *C. viridis* is a myrmecophylous beetle, *Azya orbigera* (Coccinellidae) which specializes on *C. viridis* [17]. Like many other coccinellid beetles [18], [19], [20], the larvae of *A. orbigera* are poor dispersers but are immune to ant attacks and thus experience inadvertent protection from their natural enemies in the presence of ants [21], [22]; in contrast the adult beetles are strong fliers and have the capacity to locate their prey even in small densities [23], but are vigorously attacked by the ants. Thus, adult beetles must confront physical attack by ants so as to deposit eggs in the vicinity of hemipteran insects, a task accomplished through complex behavioral mechanisms not related to the present communication and reported elsewhere [24]. In this study, we examine the role of the main specialist predator, adult *A. orbigera* beetles, in mediating hemipteran population dynamics; we additionally provide greater detail of this particular system to emphasize the key aspects relevant to the current study (Fig. 1C).

In the described research, we examine the population dynamics of ant-tended and non-tended hemipterans and investigate the potential of ant-tended patches to serve as specialist predator reservoirs for the remaining portions of the agroecosystem. Utilizing this model system, we posit that the mutualism mechanism itself critically underlies local and landscape-level pest population dynamics.

**Figure 2:**
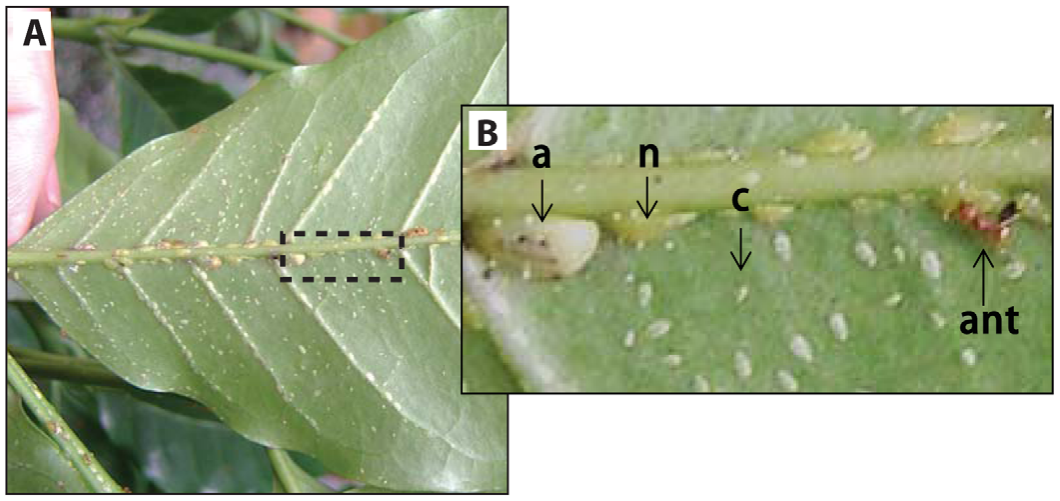
Example of digital photograph-based estimate of *C. viridis* population parameters. (A) The area of census includes the leaf area and all veins, starting from the pedicel till the 5^th^ secondary vein for the top and bottom of the abaxial surface of the leaf. (B) Close up of dashed rectangular area of leaf on day 7, where a crawler (c), nymph (n), adult (a), and ant are marked.

## Methods

The study was conducted in an organic coffee farm managed as a commercial polyculture [25], with coffee bushes growing under a canopy of species-rich shade trees (Finca Irlanda, located at 15° 11′ N, 92° 20′ W). The ant-hemipteran mutualism forms a clustered distribution, with *A. instabilis* occupying 3% of nest-sites (trees) [16], and hemipteran populations found throughout the landscape, but in greatest population density in the presence of ants [26]. Given the uniform management practices throughout the plot (in terms of light cover and soil fertility) and the lack of significant effects of abiotic factors (tree species, size, and canopy cover) on ant nest distribution, the emergent clustered pattern has been attributed to biological local interactions [16]. We conducted two field studies to examine 1) the population dynamics of the beetle *A. orbigera*, the specialist predator of the hemipteran, and 2) the mechanism of the ant-hemipteran mutualism.

The first field study investigated the population persistence of the specialist predator in a 45 ha plot, in sites with and without ants. The location of the 45 ha plot was randomly selected and the exact location of each ant nest in the plot was marked and recorded [26]. Beetles were sampled systematically by superimposing a 50×50 m grid onto the map of the plot. In each quadrant without any ant nests, beetles were sampled on coffee bushes around the centermost shade tree; in quadrants with ant nests, beetles were sampled around the shade tree with an ant nest that was closest to the center of the quadrant. In each site, we searched for adult and larvae beetles on every coffee bush within a 3 m-radius of the selected tree for 30 mins. We conducted these samplings four times over two years (rainy season 2006, dry season 2007, rainy season 2007 and dry season 2008) in 55 sites tended by *A. instabilis* and 60 sites not tended by ants. After recording adult and larval beetle populations across the four field seasons, we used a Mantel permutation test [27] to examine if beetle persistence (i.e. the proportion of times a site was occupied by at least one beetle) was correlated with ant-nest density (i.e. # of nests within a 20 m radius of the sampled tree).

**Table 1:** Estimated parameter values for Lefkovitch transitions and predation rates.

Transition probabilities & predation rates	Non-tended	Ant-tended	P-value
crawler to nymph	0.208	0.454	p<<0.001
nymph to adult	0.029	0.057	0.115
crawler predation	0.100	0.001	0.140
nymph predation	12.745	13.688	0.711
adult predation	2.200	1.402	0.274
adult fecundity	2.507	4.558	p<<0.001

**Figure 3:**
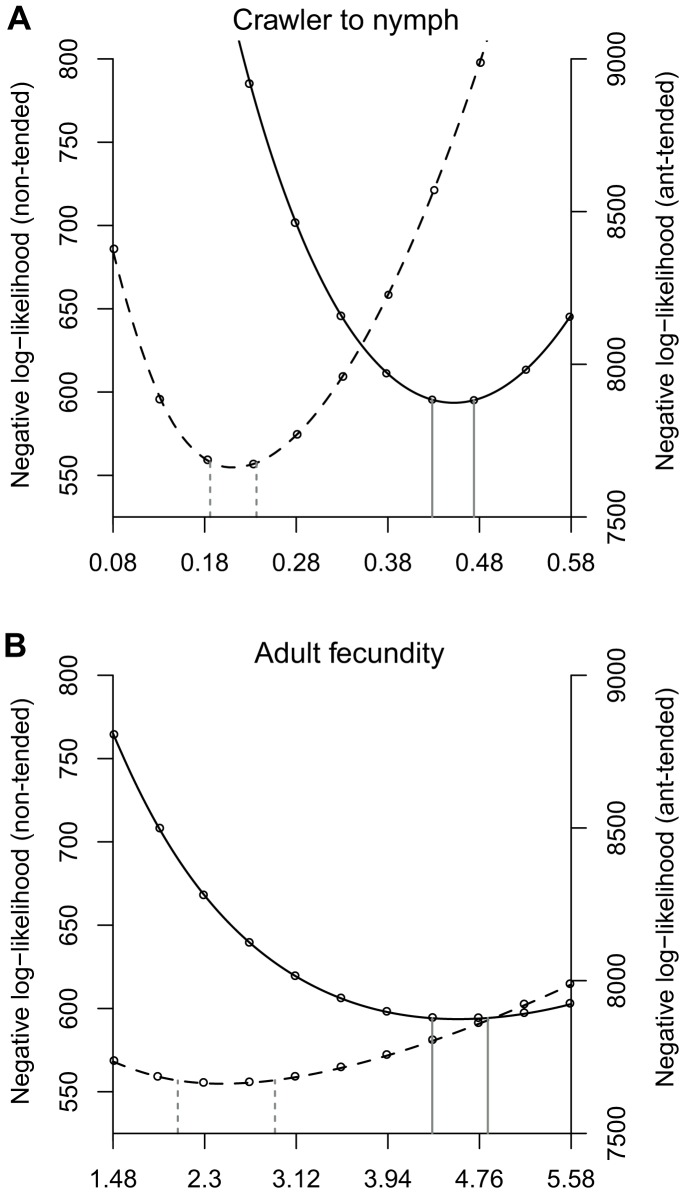
Negative log-likelihood profiles. Profiles for (A) the transition probabilities of crawler to nymph and (B) the mean adult fecundity for *C. viridus*. In both graphs, ‘ant-tended’ (solid curves) and ‘non-tended’ (dashed curves) populations are indicated. The vertical lines show the 95% confidence limits for the prediction of these probabilities calculated by the likelihood ratio test.

P-values were calculated using the likelihood ratio test. Graphical examples are indicated for the two significantly different parameters in Fig. 3.

In the second field study, we took advantage of the sessile nature of *C. viridis* by investigating hemipteran population dynamics using digital photography (Fig. 2). Photographs of the hemipteran populations were taken over two years (weekly from June to July of 2006, February to August of 2007, and July to August of 2008), and from these photos, we examined hemipteran population dynamics across three independent population cycles in ten independent randomly chosen study sites (separated by more than 500 m), six of which were closely associated with a nest of the arboreal nesting *A. instabilis* and four of which were in an area known to be at least 200 m removed from any such nest (*N = 30* populations, *N = 8–30* weekly time steps per population) [28]. _ENREF_29Using the photographs, we counted the number of intact and predated crawlers, nymphs, and adults each week on each leaf, in sites with and without ants. The latter was possible by comparing consecutive pictures and counting newly and previously predated hemipterans; the process was further enabled by the fact that the main predator, *A. orbigera*, leaves telltale feeding marks on predated hemipterans [28]. By directly measuring predation on the scales, we document differential predation rates by predators across ant-tended and non-tended sites.

Using the counts of individuals and predation events, we built a simple Leftkovitch stage-based model [29], [30] to describe *C. viridis* population dynamics. The model has six parameters: crawler-to-nymph and nymph-to-adult transition probabilities; crawler, nymph, and adult predation parameters; and mean adult fecundity. Unlike traditional Leftkovitch models our model has density dependence, with the predation probability dependent on the population number of a leaf. The predation probability for a given life stage on a given leaf for a given predation parameter is equal to:
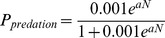
where *N* is the population of scales of that life stage on that leaf and *a* is the predation parameter. For a given set of parameters we calculated the likelihood of observing our ant-tended and non-tended *C. viridis* time series, and we searched for two sets of parameters that maximized these likelihoods using the *fminsearch* function in Matlab [31]. For each parameter we created likelihood profiles to see how sensitive the likelihood was to a change in that parameter [31]. To create a likelihood profile, a given parameter is fixed at one of a set of values and then the likelihood is maximized by letting the other parameters vary, again using *fminsearch*. The result is a curve showing the likelihood of the model predicting the data given a fixed value of one of the parameters. This was repeated for each of the six parameters, and for ant-tended and non-tended populations. As is convention, we plot the negative log-likelihood, so a smaller value is more likely. We then used the likelihood ratio test to determine which parameters were significantly different between ant-tended and non-ant-tended sites [32].

## Results

Based on the first field study, our results indicate that both adult beetle persistence (*P = 0.002*) and larvae beetle persistence (*P = 0.004*) were highly correlated with ant nest density (Mantel test, *N = 115*). Neither adult nor larvae beetle persistence was spatially auto-correlated (Moran’s I, *N = 115, P>0.411* for both). Results from the second field study (Table 1) indicate that hemipteran nymph and adult predation rates were not significantly different between ant-tended and non-tended sites. Instead, the hemipteran transition probability from crawler to nymph was significantly higher in the presence of ants (Fig. 3A) and hemipteran adult fecundity was significantly higher in the presence of ants (Fig. 3B) (Table 1).

## Discussion

In this field-based study, we show 1) that the mutualism patches are indeed spatial reservoirs for the specialist predator, *A. orbigera*, and 2) that predation rates on the hemipterans are equally high in ant-tended and non-tended patches. Our results indicate that the specialist predator is able to prey equally well inside and outside ant-tended patches. Accordingly, independent studies show that the protective behavior of the ant is effectively cancelled when the ant is under attack by a phorid parasitoid, and *A. orbigera* is then able to oviposit near and prey upon ant-tended scale colonies [23], [33]. However, due to the direct path of the ant-hemipteran mutualism, *C. viridis* populations grow faster inside the ant-tended areas, creating spatial reservoirs for *A. orbigera*, which then potentially exert effective biological control over the rest of the farm. Thus we propose that the direct nature of the mutualistic mechanism (Fig. 1) simultaneously benefits and limits hemipteran population growth, depending on the spatial scale of the interaction.

Effective control of hemipteran populations in this system is a consequence of the self-organized heterogeneity of the ant-hemipteran mutualism [17], [21], [22]. While studies conducted across taxa have suggested that mutualisms may be exploited by specialist predators [34], [35], the relative cost or benefit of ant-protection to hemipteran populations has not been well-established [9]. Our results indicate that while specialist predator persistence was significantly higher in ant-tended patches, hemipteran predation rates were not significantly different between the patches. Ant-presence has no effect on predation rates likely because the specialist predator larvae is immune to ant attacks and also experiences inadvertent protection from its natural enemies in the presence of ants [21], [22]. Thus, in this system, we show that if ants are providing hemipterans protection from predation, this protection is only enough to balance increased predation pressure in ant-tended sites.

Most importantly, our results clearly demonstrate that ants provide a benefit to hemipterans, but that the mutualism occurs via enhanced *C. viridis* crawler to nymph transition probabilities and increased adult fecundity, not protection from predation. Other than protection from predation, the two other benefits of ant-tending hypothesized in past studies, are the 1) increased hygienic conditions for hemipterans via the removal of hemipteran honeydew [11], and 2) better site-selection in ant-tended populations, leading to increased nutrient uptake [36]. The majority of past studies suggest that hemipterans may actually incur a reduction in fecundity in the presence of ants [37], potentially due to a forced increase in honeydew production [38] and/or reduced assimilation of N by hemipterans [9], [39], [40]. In contrast, our study demonstrates that the primary benefit ant-tended hemipterans experience is increased fecundity and growth rates, a finding that has been documented only in cage experiments in the absence of natural enemies [36]. While the exact ant-mediated activity leading to the growth-based mutualism is not known, we hypothesize that it may be due to a combination of improved hygienic conditions and feeding site selection mediated by ants.

Given the strong dependence of natural and agricultural systems on mutualisms, pest control decisions made in the light of incorrect assumptions about the mechanism of the mutualism could lead to predator population decline and loss of this critical ecosystem function. In this study, we provide evidence that the ant-hemipteran mutualism is driven by the direct enhancement of hemipteran growth rates and fecundity. Based on the data, we posit that this occurs via the creation of spatial reservoirs for specialist hemipteran predators. Therefore, in agroecosystems where ants tend hemipterans, the recommendation to eliminate ants for pest control may be counterproductive. If, as in this study, the direct mutualism mechanism supports predators that control hemipteran pests at the general level of the farm, then the mutualism itself must be thought of as part of the biological control function and be preserved.

## References

[1] Vandermeer J (1995) The ecological basis of alternative agriculture. Annual Review of Ecology and Systematics 26: 201–224.

[2] Klein AM, Vaissiere BE, Cane JH, Steffan-Dewenter I, Cunningham SA, et al (2007) Importance of pollinators in changing landscapes for world crops. Proceedings of the Royal Society B-Biological Sciences 274: 303–313. 10.1098/rspb.2006.3721 PMC1702377 17164193

[3] Freiberg C, Fellay R, Bairoch A, Broughton WJ, Rosenthal A, et al (1997) Molecular basis of symbiosis between Rhizobium and legumes. Nature 387: 394–401. 9163424 10.1038/387394a0

[4] Brussaard L, de Ruiter PC, Brown GG (2007) Soil biodiversity for agricultural sustainability. Agriculture Ecosystems & Environment 121: 233–244.

[5] Way M (1963) Mutualism between ants and honeydew-producing homoptera. Annual Review of Entomology 8: 307–344.

[6] Buckley RC (1987) Interactions involving plants, homoptera, and ants. Annual Review of Ecology and Systematics 18: 111–135.

[7] Sogawa K (1982) The rice brown planthopper - feeding physiology and host plant interactions. Annual Review of Entomology 27: 49–73.

[8] Ragsdale DW, Voegtlin DJ, O’Neil RJ (2004) Soybean aphid biology in North America. Annals of the Entomological Society of America 97: 204–208.

[9] Stadler B, Dixon AFG (2005) Ecology and evolution of aphid-ant interactions. Annual Review of Ecology Evolution and Systematics 36: 345–372.

[10] Haines I, Haines J (1978) Pest status of the crazy ant Anoplolepis longipes (Hymenoptera, Formicidae) in the Seychelles. Bulletin of Entomological Research 68: 627–638.

[11] Fokkema N, Riphagen I, Poot R, de Jong C (1983) Aphid honeydew, a potential stimulant of Cochliobolus satirus and Septoria nodorum and the competitive role of saprophytic mycoflora. Transactions British Mycological Society 81: 355–363.

[12] Banks CJ, Macaulay ED (1967) Effects of *Aphis Fabae Scop* and of its attendant ants and insect predators on yields of field beans (*Vicia faba* L). Annals of Applied Biology 60: 445–&.

[13] Harmon JP, Andow DA (2007) Behavioral mechanisms underlying ants’ density-dependent deterrence of aphid-eating predators. Oikos 116: 1030–1036.

[14] Nault LR, Montgomery ME, Bowers WS (1976) Ant-aphid association - role of aphid alarm pheromone. Science 192: 1349–1351. 1273595 10.1126/science.1273595

[15] Oliver TH, Jones I, Cook JM, Leather SR (2008) Avoidance responses of an aphidophagous ladybird, Adalia bipunctata, to aphid-tending ants. Ecological Entomology 33: 523–528.

[16] Vandermeer J, Perfecto I, Philpott SM (2008) Clusters of ant colonies and robust criticality in a tropical agroecosystem. Nature 451: 457–U453. 18216853 10.1038/nature06477

[17] Liere H, Perfecto I (2008) Cheating on a mutualism: Indirect benefits of ant attendance to a coccidophagous coccinellid. Environmental Entomology 37: 143–149. 18348805 10.1603/0046-225x(2008)37[143:coamib]2.0.co;2

[18] Volkl W (1995) Behavioral and Morphological Adaptations of the Coccinellid, Platynaspis-Luteorubra for Exploiting Ant-Attended Resources (Coleoptera, Coccinellidae). Journal of Insect Behavior 8: 653–670.

[19] Volkl W, Vohland K (1996) Wax covers in larvae of two Scymnus species: Do they enhance coccinellid larval survival? Oecologia 107: 498–503. 28307393 10.1007/BF00333941

[20] Bartlett B (1961) The influence of ants upon parasites, predators, and scale insects. Annual Entomological Society of America 54: 543–551.

[21] Perfecto I, Vandermeer J (2008) Spatial pattern and ecological process in the coffee agroforestry system. Ecology 89: 915–920. 18481515 10.1890/06-2121.1

[22] Vandermeer J, Perfecto I, Philpott S (2010) Ecological Complexity and Pest Control in Organic Coffee Production: Uncovering an Autonomous Ecosystem Service. Bioscience 60: 527–537.

[23] Liere H, Larsen A (2010) Cascading trait-mediation: disruption of a trait-mediated mutualism by parasite-induced behavioral modification. Oikos 119: 1394–1400.

[24] Hsieh H, Perfecto I (2012) Trait-mediated indirect effects of phorid flies on ants. Psyche: A Journal of Entomology 2012: 1–11.

[25] Moguel P, Toledo VM (1999) Biodiversity conservation in traditional coffee systems of Mexico. Conservation Biology 13: 11–21.

[26] Vandermeer J, Perfecto I (2006) A keystone mutualism drives pattern in a power function. Science 311: 1000–1002. 16484494 10.1126/science.1121432

[27] Mantel N (1967) The detection of disease clustering and a generalized regression approach. Cancer Research 27: 209–220. 6018555

[28] Jha S, Vandermeer J, Perfect I (2009) Population dynamics of Coccus viridis, a ubiquitous ant-tended agricultural pest, assessed by a new photographic method. Bulletin of Insectology 62: 183–189.

[29] Lefkovitch L (1965) Study of population growth in organisms grouped by stages. Biometrics 21: 1–18.

[30] Caswell H (2001) Matrix Population Models: Construction, Analysis, and Interpretation. Sunderland, MA: Sinauer Associates. 722 p.

[31] MATLAB (2003) Version 6.5.1. Natick, Massachusetts: The MathWorks Inc.

[32] Bolker BM (2008) Likelihood and all that. Ecological Models and Data in R: Princeton University Press. 169–221.

[33] Hsieh H, Liere H, Soto EJ, Perfecto I (in press) Cascading trait-mediated interactions induced by ant pheromones. Ecology and Evolution. 10.1002/ece3.322 PMC3488669 23139877

[34] Bronstein JL (2001) The exploitation of mutualisms. Ecology Letters 4: 277–287.

[35] Wimp GM, Whitham TG (2001) Biodiversity consequences of predation and host plant hybridization on an aphid-ant mutualism. Ecology 82: 440–452.

[36] Banks CJ (1958) Effects of the Ant, *Lasius niger* (L.), on the Behaviour and Reproduction of the Black Bean Aphid, *Aphis fabae* Scop. Bulletin of Entomological Research Supplement Series 49: 701–714.

[37] Yao I, Shibao H, Akimoto S (2000) Costs and benefits of ant attendance to the drepanosiphid aphid Tuberculatus quercicola. Oikos 89: 3–10.

[38] Nixon G (1951) The Association of Ants with Aphids and Coccids. London: Commonwealth Inst. Entomol. 36 p.

[39] Stadler B, Dixon AFG (1998) Costs of ant attendance for aphids. Journal of Animal Ecology 67: 454–459.

[40] Stadler B, Dixon AFG, Kindlmann P (2002) Relative fitness of aphids: effects of plant quality and ants. Ecology Letters 5: 216–222.

